# Establishing In Vitro Dosimetric Models and Dose–Effect Relationships for ^177^Lu-DOTATATE in Neuroendocrine Tumors

**DOI:** 10.2967/jnumed.125.269470

**Published:** 2025-08

**Authors:** Giulia Tamborino, Pleun Engbers, Tijmen H. de Wolf, Thom G.A. Reuvers, Rob Verhagen, Mark Konijnenberg, Julie Nonnekens

**Affiliations:** 1 Department of Radiology and Nuclear Medicine, Erasmus MC Cancer Institute, Erasmus University Medical Center, Rotterdam, The Netherlands; and; 2 Department of Molecular Genetics, Erasmus MC Cancer Institute, Erasmus University Medical Center, Rotterdam, The Netherlands

**Keywords:** cellular dosimetry, *S* values, peptide receptor radionuclide therapy, relative biologic effectiveness, neuroendocrine tumors

## Abstract

This study investigates the radiobiology of peptide receptor radionuclide therapy (PRRT) using clinically relevant cancer cell lines by developing a framework for realistic cellular dosimetry in 2- and 3-dimensional cluster-forming configurations. **Methods:** The radiobiologic responses of GOT1 and NCI-H69 tumor cell lines to PRRT and external beam radiotherapy (EBRT) were compared. Viability at 7 d and cell death at multiple time points were assessed. Image-based multicellular dosimetry models were developed to reflect in vitro exposure complexity and were compared with traditional approaches. **Results:** The PRRT absorbed dose in suspension was dominated by medium during incubation and by a cross-dose within small clusters after incubation. Our findings reveal that traditional dosimetry can underestimate absorbed doses by up to 90% in plated setups and overlooks dose heterogeneity, with initial dose rates varying by up to 2.3-fold based on cluster size and cell arrangement. The maximum relative biologic effectiveness of PRRT compared with EBRT for loss of viability at 7 d was 0.43 ± 0.07 for NCI-H69 cells and 0.22 ± 0.02 for GOT1 cells. NCI-H69 cells showed greater resistance to PRRT-induced cell death than to EBRT, whereas GOT1 cells exhibited similar cell death levels for both treatments, albeit with different dose–response dynamics. **Conclusion:** PRRT requires on average an absorbed dose 3 times higher than EBRT to achieve equivalent effects in vitro. Traditional dosimetry overestimates the relative biologic effectiveness by underestimating the absorbed dose.

## Visual Abstract

**Figure:**
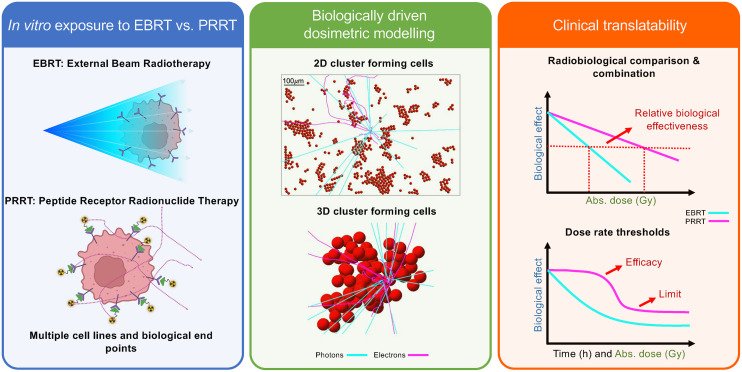

Peptide receptor radionuclide therapy (PRRT) is a systemic targeted radionuclide therapy that has shown considerable promise in treating solid tumors and micrometastases (1). PRRT is used for inoperable neuroendocrine tumors (NETs) overexpressing somatostatin receptor type 2 (2) using [^177^Lu]Lu-DOTA-Tyr^3^-octreotate (^177^Lu-DOTATATE), which has demonstrated significant improvements in survival and quality of life in patients over alternative treatments (3,4).

To better understand the potential of PRRT, its clinical optimization process can be compared with other radiation-based treatments, such as widely used external beam radiotherapy (EBRT). Optimization of radiation-based treatments relies on evaluating absorbed dose–effect relationships in preclinical and clinical studies to ensure both efficacy and safety.

However, in vitro PRRT studies often report biologic effects in relation to the administered activity or provide absorbed dose estimates based on simplified models of the cellular absorbed dose. These simplifications fail to capture the complexity of the biologic experiments performed to evaluate the relevant biologic endpoint, potentially leading to inaccurate biophysical correlations and interpretations.

Recent studies have demonstrated a clear dose–response relationship in grade 2 NETs treated with ^177^Lu-DOTATATE (5–7). However, accurate dosimetry in clinical settings remains challenging because of the limited resolution (in millimeters) of clinical imaging to measure radionuclide activity and the inherent dosimetric uncertainties for small tumor volumes (<5 cm^3^). These challenges are particularly significant for PRRT, because accurately assessing the distribution of radionuclides is crucial for determining treatment efficacy.

In addition, both physical heterogeneity (e.g., absorbed dose and dose rate) and biologic heterogeneity (e.g., radiosensitivity, repair, redistribution, and reoxygenation), which are likely more pronounced in larger tumors, influence tumor responses, hindering the establishment of clear dose–response relationships for PRRT.

Radiobiologic models, typically derived from EBRT, may not directly translate to PRRT because of differences in timing, dose rate, and exposure uniformity, complicating the identification of clear PRRT dose–response relationships in clinical settings (5).

Preclinical in vitro and in silico models can help elucidate the role of absorbed dose heterogeneity at cellular and subcellular levels, overcoming the limitations of clinical imaging modalities. Furthermore, considering dose rate effects, specific radiosensitivity, and repair parameters is essential to the development of well-tailored PRRT regimens. These considerations are also vital for exploring potential combinations with other therapeutic approaches to increase treatment efficacy.

In this work, we aimed to address these gaps by providing specific radiobiologic parameters for PRRT using clinically relevant cancer cell lines, including fast- and slow-proliferating models. In addition, we established a framework for accurate cellular dosimetry calculations in 2- and 3-dimensional cluster-forming scenarios during radionuclide exposure. By improving our understanding of the biologic effectiveness of PRRT, we hope to support the development of optimized treatment strategies.

## MATERIALS AND METHODS

### Cell Lines and Treatment

Experiments were performed with the somatostatin receptor type 2–positive human small cell lung cancer cell line NCI-H69, which grows in suspension, and the NET cell line GOT1 (Supplemental Fig. 1), which is adherent (supplemental materials are available at http://jnm.snmjournals.org). More information on origin and culture conditions is in the supplemental materials.

For uptake, viability, and cell death experiments, cells were treated with different activity concentrations of ^177^Lu-DOTATATE (molar activity, 53 MBq/nmol; peptide mass, 1.51–30.19 ng/mL; radiochemical yield, >98%; radiochemical purity, >95%) or x-ray treatment (at 86 keV) at a constant dose rate of 1.6 Gy/min, as detailed in the supplemental materials (8) and summarized here.

### Uptake Assay and Assumptions

Membrane-bound and internalized fractions were collected for different activity concentrations of ^177^Lu-DOTATATE (GOT1, 0.05, 0.25, 1 MBq/mL; NCI-H69, 0.1, 0.25, 1 MBq/mL) as described in the supplemental materials. Fractions of added activity were determined for activities of 0.1 or 0.05 MBq/mL, depending on the cell line, and 0.25 MBq/mL by 3-dimensional inverse distance weighting extrapolation using Python (Python Software Foundation).

The uptake and excretion data for both cell lines were fitted with the least squares method to a monoexponential uptake and a monoexponential decay followed by a plateau, respectively. Membrane-bound and internalized activity were assumed to be uniformly distributed among the cell population and equally divided among proliferating cells. Details on how heterogeneous activity distributions were modeled (Supplemental Figs. 1 and 2), along with the proliferation assay, are provided in the supplemental materials.

### Viability Assay

For PRRT, cells were treated in suspension with an activity concentration range (0.05, 0.1, 0.25, 0.5, or 1.0 MBq/mL or vehicle) for 4 h in 1 mL of culture medium (2 × 10^5^ cells/mL). For EBRT, cells were irradiated (GOT1, 0.5, 1, 2, 4 Gy; NCI-H69, 1, 2, 3, 4 Gy) under similar experimental conditions (brief EBRT exposure of cells in suspension a 2 × 10^5^ cells/mL after 4 h of incubation). After irradiation, cells were washed and plated (with 40,000 cells/mL for NCl-H69 and 150,000 cells/mL for GOT1) in triplicate, and viability was measured at 7 d, as reported in the supplemental materials.

### Cell Death Assay

For EBRT, GOT1 cells were seeded 3 d before irradiation into 6-well plates. NCI-H69 cells were seeded directly before irradiation into 100-mm cell-culture dishes. Cells were irradiated with 0.25, 0.5, 1, or 2 Gy, and cell death was measured at 1, 4, 7, 11, and 14 d after treatment. For PRRT, cells were treated in suspension with an activity concentration range (0.1, 0.25, 0.5, or 1 MBq/mL or vehicle) for 4 h in 10 mL of culture medium (2 × 10^5^ cells/mL) and seeded into 6-well plates (GOT1) or 100-mm cell-culture dishes (NCI-H69) at densities similar to those used in the viability assays. Cell death was measured at 4, 7, 11, and 14 d after treatment. Cell death detection was performed using the Zombie NIR Fixable Viability Kit (BioLegend). The fluorescent signal was detected by flow cytometry on a LSRFortessa flow cytometer (BD Biosciences). Gating and data analysis were performed using FlowJo software (BD Biosciences). Experiments were performed as 3 independent (technical) replicates.

### Microscopic Image Collection, Analysis, and Data Conversion

Immunofluorescent staining confirmed the spheric morphology of both cell lines (Supplemental Fig. 3), and an automated circle detection algorithm determined the cellular radii (GOT1, 5.5 ± 1.1 µm; NCI-H69, 7.1 ± 1.5 µm) and nuclear radii (GOT1, 5.0 ± 0.5 µm; NCI-H69, 4.1 ± 0.6 µm). Cell membrane thickness was set to 7.5 nm (9).

Brightfield images preserving the cellular density used in viability and cell death assays were collected for both cell lines at various magnifications and time points (Supplemental Figs. 4 and 5). Floating cells were centrifuged to the bottom of the well, whereas attached cells were imaged directly. Data analysis, including edge detection, cluster differentiation, and spatial analyses, was used to inform realistic cellular setups for floating and plated cells. Sample images and analysis methods are detailed in the supplemental materials.

### Simulations of Absorbed Dose per Decay

**FIGURE 1.**
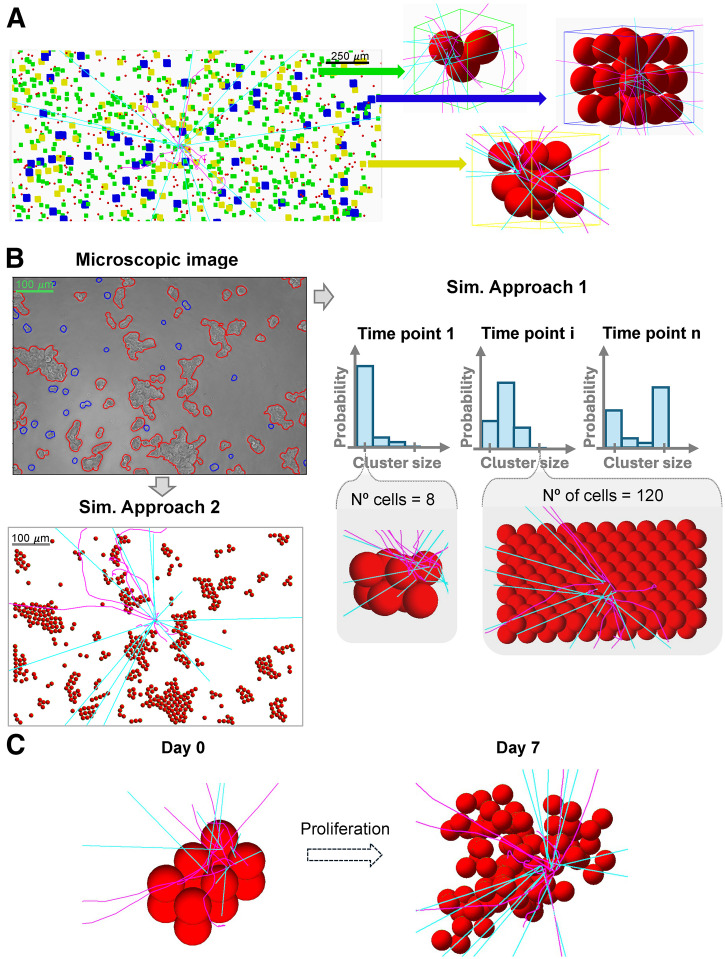
*S* value simulation setups for GOT1 and NCI-H69 cells using Geant4. (A) Floating GOT1 clusters during 4-h uptake phase modeled with random close packing, with zoomed-in views on right. (B) Microscopic image of GOT1 cells plated for 7-d follow-up phase (top left) modeled with cluster size probabilities (top right) and hexagonal packing (bottom right) or microscopic image-derived placements (bottom left). (C) Simulated cluster geometry for NCI-H69 cells in suspension at day 0 (compact, N° = 12) and day 7, illustrating exponential growth and random placement after proliferation. Sim. = simulated.

Simulations of the absorbed dose per decay (*S* value) were conducted using Geant4 (Geant4 Collaboration) (10), with details on source, material, and physics settings provided in the supplemental materials. For GOT1 cells, simulations were divided into 2 stages: a 4-h uptake phase in radioactive medium suspension (Fig. 1A) followed by a 7-d plated incubation phase (Fig. 1B). In contrast, for NCI-H69 cells, both the uptake and the incubation phases occurred in suspension (Fig. 1C).

#### GOT1 Uptake Phase (Floating Cells)

A random close packing algorithm based on disciplined convex–concave programming (11) was used to model clusters of variable sizes (2–60 cells), with maximization of the cellular packing fraction depending solely on the number of cells. Cellular positions were input into Geant4 to simulate floating cellular geometries. Cells at cluster centers and edges were automatically detected and used as the target to score the *S* value in their nuclei from membrane and cytoplasm sources. The separate contributions of electron and γ-radiation was recorded.

The frequency of cluster size formation, assuming spheric symmetry, was used to weight the total cross-absorbed dose delivered by variable-sized clusters to the target cell, evaluating the average *S* value within the cluster to which the target cell belongs (the in-cluster contribution). Next, a cylinder sized as the maximum β-range of ^177^Lu particles (12) was filled with single cells and clusters of 3, 12, and 28 cells, according to fixed probabilities (70%, 20%, and 10%, respectively) up to a set volume fraction (<1%) based on the collected experimental data (Fig. 1A). Positions and sizes of cells and clusters were used in the simulation geometry to account for absorbed dose contributions from surrounding cells and clusters (cross-cluster contribution). The absorbed dose contribution from the medium was evaluated by simulating a cylindric 10-mL tube.

#### GOT1 Follow-up (Plated Cells)

The plated cells were modeled using 2 approaches: cells were placed according to an ideal hexagonal face-centered packing algorithm without space between them, thereby neglecting any cross-cluster contribution (Fig. 1B, right), or cells were placed using 8 masked microscopic images of plated cells as maps in 3ds Max software (Autodesk), thereby reproducing the setups as realistically as possible (Fig. 1B, left). In both cases, clusters were assumed to be made of 2 layers, according to available nucleus-stained images.

For the first approach, simulations similar to the floating case were performed, weighting the in cluster cross-absorbed dose by cluster size frequency and analyzing the variability in the average absorbed dose between a target cell localized at the edge versus the center of the cluster. For the second approach, 10 target cells were randomly selected to include various cluster sizes and single cells. The number of setups and targets was based on data variability.

#### NCI-H69 Uptake and Follow-up (Floating Cells)

NCI-H69 cell clusters were modeled starting from initial close-packed clusters of cells (N_0_ = 3–37 cells; N_0_ is the number of cells at the start of the simulation), using an exponential growth pattern (Fig. 1C) with a doubling time of 58.4 ± 0.7 h (Supplemental Fig. 6). New cells were added near randomly chosen parent cells each day for 7 d. For the initial cell clusters and each day, cell positions were recorded and used as the inputs for simulations. The target cell was the centrally placed seeding cell.

Simulations for each initial cell cluster and days 0–7 were run multiple times (i.e., 35–50 runs) until convergence, reducing uncertainty from random cell placement. The total cross-*S* value for each day was determined using cluster size frequency from day 0. Alternatively, cell numbers for each day were matched to corresponding tabulated *S* values to weight the cross-*S* value by actual cluster size frequencies on days 1, 3, 4, and 6, evaluating the effect of cell clumping on the *S* value.

### Absorbed Dose Calculations

MIRD formalism (13) was used to combine the simulated *S* values with activity measurements to evaluate absorbed dose rates during the cell death and viability assays. For GOT1 cells, the dynamic transition between the uptake (3-dimensional floating) and the decay (2-dimensional plated) phases was modeled using a logistic function.

The absorbed dose was calculated by numerically integrating the absorbed dose rates over the time range corresponding to the biologic endpoint assessment. Details on dynamic transition modeling, error propagation, and statistical analysis are provided in the supplemental materials.

### Absorbed Dose–Response Correlations

The correlation between absorbed dose and cell viability at day 7 for both EBRT and PRRT was fitted using either a linear quadratic or a linear model. The relative biologic effectiveness (RBE) was determined from α-ratios derived via linear fitting.

The relationship between the fraction of alive cells and either time or the absorbed dose was modeled for PRRT using a logistic function (Eq. 1), with *F*_min​_ as the minimum fraction, *k* as the death rate constant, and *T*_0_ as the inflection point (i.e., the lag time when significant cell death is detected):

$$F(t)=F_{\min}+\frac{\left(1-F_{\min}\right)}{\left(1+e^{\left(k\;(t-T_{0}\right)}\right)}$$ Eq. 1

For EBRT, *F*(*t*) was modeled as an exponential decay with regrowth (Eq. 2), where *k* is the death rate constant, *k*_0​_ is the regrowth rate constant, *T*_0_​ is the transition time, and *S*(*t*) is the smoothing function:

$$F\left(t\right)=e^{-k_{0}t}\left(1-S\left(t\right)\right)+{e^{-k_{0}t}\;e}^{k_{0}\left(t-T_{0}\right)}\;S(t)$$ Eq. 2

## RESULTS

### Higher Internalized Activity in NCI-H69 Cells Compared with GOT1

**FIGURE 2.**
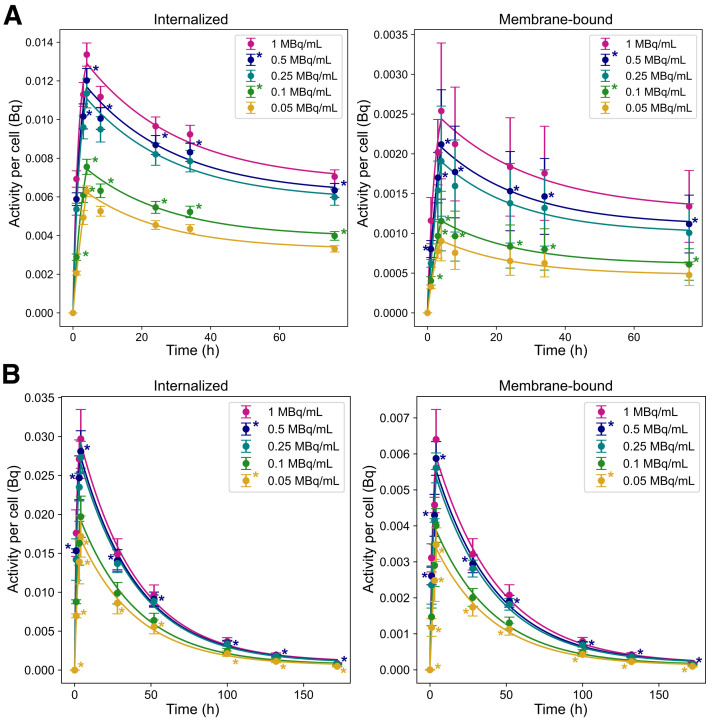
Time-dependent activity per cell for GOT1 (A) and NCI-H69 (B) cells at various activity concentrations. Asterisks indicate extrapolated data points. Error bars indicate SD.

The internalized and membrane-bound time–activity curves are shown in Figure 2 and Supplemental Table 1. The average effective half-life (i.e., cellular excretion rate) for NCI-H69 and GOT1 cells is 22.6 ± 0.15 and 16.4 ± 0.40 h, respectively, as determined from the exponential fit of the data shown in Figure 2.

### Medium and In-Cluster Cross-Dose as Primary Dose Contributors During Incubation

During the 4-h uptake phase for GOT1 cells, the medium accounted for 70% of the absorbed dose, with in-cluster contributions (including a single-cell self-dose) making up 27% (Supplemental Fig. 7). Cross-cluster and single-cell contributions were negligible (<2%). At the highest activity concentration (1 MBq/mL), the absorbed dose after 4 h was similar at the cluster center (0.48 ± 0.02 Gy) and edge (0.45 ± 0.01 Gy) because of the small cluster size and limited uptake.

For NCI-H69 cells, randomly arranged floating clusters were modeled in 3 dimensions, with the cross-dose increasing with cluster size but plateauing for clusters larger than approximately 20 cells (Supplemental Fig. 8). The random placement of cells reduced cross-dose contributions, and by days 3–4, the predominance of small clusters and the fast effective decay rate rendered the dose rate largely inefficacious (Supplemental Table 2). Additional details, including self-absorbed dose *S* values, in-cluster cross-dose correlations, time-dependent *S* value correlations parameterized by initial cluster size, and generalized formulas for the growth-dependent absorbed dose, are provided in the supplemental materials.

### Cross-Cluster Contributions and Dose Rate Heterogeneity in GOT1 Plated Phase

**FIGURE 3.**
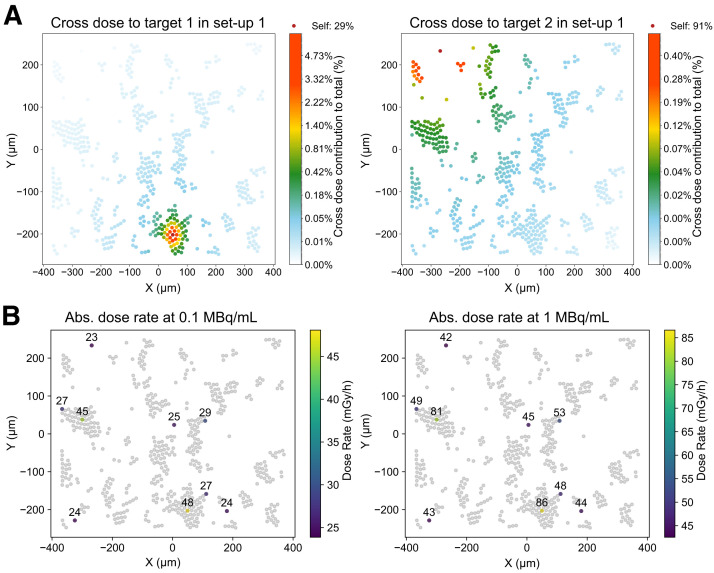
Cross-dose variability and absorbed dose rate heterogeneity in GOT1 cells. (A) Cross-dose contribution relative to total for 2 target cells within same setup. (B) Absorbed dose rate maps (excluding medium contribution) for 10 target cells at start of incubation at 0.1 and 1 MBq/mL. Abs. = absorbed.

During incubation, GOT1 cells formed bilayer clusters and single cells adhering to the plating surface. Dosimetric modeling informed by realistic cellular placement (Fig. 1B) revealed average total cross-*S* values of 2.37 ± 1.50 Gy/Bq/h for the membrane and 2.36 ± 1.49 Gy/Bq/h for the cytoplasm, whereas the hexagonal packing model underestimated the cross-dose by 66%–90% (Supplemental Fig. 9). Details on the in-cluster contribution of the hexagonally packed bilayer are provided in the supplemental materials. Figure 3A highlights the significant variability in the cross-dose compared with a self-dose, driven by the specific cellular configuration and placement.

Dose rate heterogeneity resulted in up to +77% and −24% variation at the start of incubation (Fig. 3B; Supplemental Table 3). A bimodal dose distribution better captured the variability between single or small clusters and larger clusters (Supplemental Fig. 10), with both subpopulations showing a strong negative correlation (*r* = −0.96) between absorbed dose and cell viability, supporting the use of the average absorbed dose for viability correlations.

### Dose Rate-Dependent Survival Trends in GOT1 Cells

**FIGURE 4.**
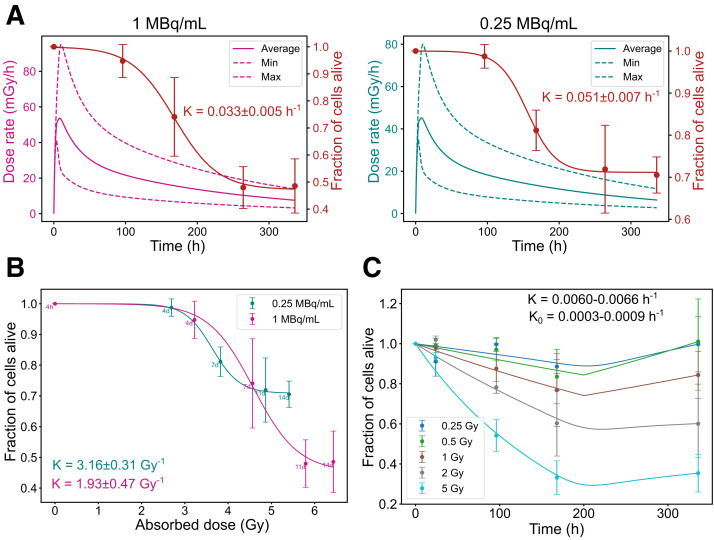
Radiobiologic correlations for GOT1 cells. (A) Time-dependent dose rate (left, *y*-axis) and fraction of alive cells (right, *y*-axis) at 1 and 0.25 MBq/mL. (B) Correlation between absorbed dose and fraction of alive cells for same added activities. (C) Fraction of alive cells over time for EBRT exposure. Error bars indicate SD. Dashed lines indicate dose rate heterogeneity. K = death rate constant; K_0​_ = regrowth rate constant.

Dosimetric parameters (maximum and minimum dose rates and average absorbed dose) and biologic parameters for GOT1 cell survival are summarized in Supplemental Table 4. Higher initial dose rates resulted in a more gradual and sustained decline in the fraction of alive cells. In contrast, lower dose rates resulted in a sharp initial drop in cell survival, followed by stabilization at higher survival levels over time. This trend was evident in both the time correlation (Fig. 4A) and the absorbed dose correlation (Fig. 4B). Under EBRT, cell death followed a conventional dose-dependent pattern, with higher absorbed doses causing greater cell death, followed by regrowth (Fig. 4C).

### NCI-H69 Cells: Resistance to PRRT and Rapid Regrowth After EBRT

**FIGURE 5.**
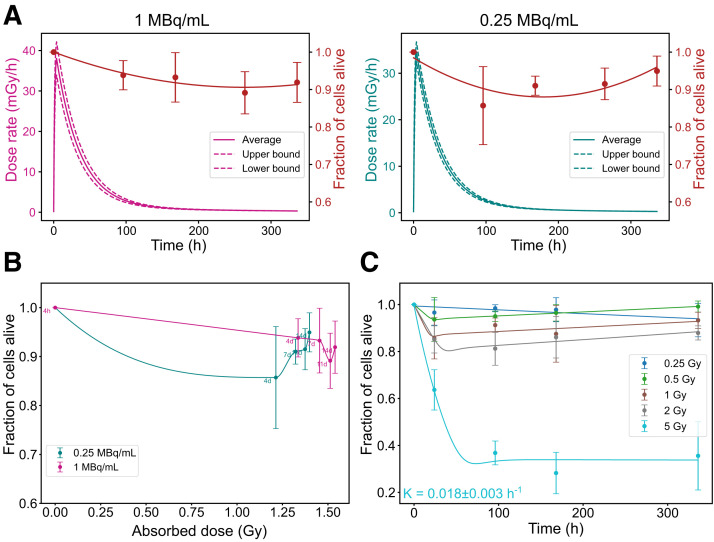
Radiobiologic correlations for NCI-H69 cells. (A) Time-dependent dose rate (left, *y*-axis) and fraction of alive cells (right, *y*-axis) at 1 and 0.25 MBq/mL. (B) Correlation between absorbed dose and fraction of alive cells for same added activities. (C) Fraction of alive cells over time for EBRT exposure. Error bars indicate SD. Dashed lines indicate dose rate heterogeneity. K = death rate constant.

Dosimetric parameters for NCI-H69 cells are presented in Supplemental Table 5. Unlike GOT1 cells, NCI-H69 cells exhibited minimal reduction in the fraction of alive cells regardless of the dose rate, with only slight differences observed between higher and lower dose rates (Fig. 5A). Across all activity concentrations, the minimum fraction of alive cells stabilized around 0.88 ± 0.04. This minimum was reached earlier at lower activity levels, likely due to reduced initial cell death, which allowed for quicker onset of regrowth, as shown in both the time correlation and the absorbed dose correlation (Fig. 5B). Under EBRT, NCI-H69 cells exhibited a conventional dose-dependent response, with significant cell death occurring only at the highest absorbed dose (5 Gy). However, unlike GOT1 cells, NCl-H69 cells rapidly resumed proliferation almost immediately after exposure (Fig. 5C).

### Dose–Response Parameters and RBE

**FIGURE 6.**
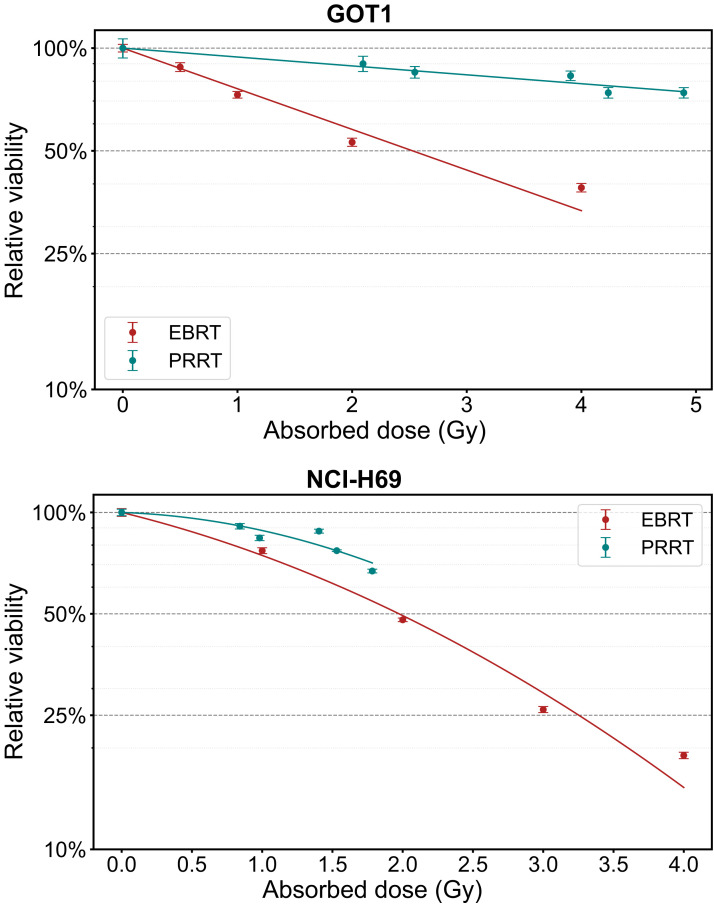
Dose–response curves showing absorbed dose vs. viability at 7 d for GOT1 and NCI-H69 cells exposed to EBRT or PRRT. Error bars indicate SD.

At day 7, the average absorbed dose ranged from 0.98 to 1.78 Gy for NCI-H69 cells and from 2.55 to 4.89 Gy for GOT1 cells, including the medium contribution (Supplemental Table 6). The correlation between the absorbed dose and the relative decrease in cell viability is depicted in Figure 6, with the corresponding best fitting parameters and linearized results for maximum RBE evaluation detailed in Table 1. The maximum RBE for decrease in viability, based on a linear dose response, was 0.22 ± 0.02 for GOT1 cells and 0.43 ± 0.07 for NCI-H69 cells compared with EBRT.

**TABLE 1.** Summary of Best Fitting Parameters Used for Dose Responses Shown in Figure 6 After Exposure of GOT1 and NCI-H69 Cells to EBRT and PRRT

	NCI-H69					GOT1		
	LQ					L		
Exposure	*R* ^2^	AIC	α (Gy^−1^)	β (Gy^−2^)	Linearized α (Gy^−1^)	*R* ^2^	AIC	α (Gy^−1^)
EBRT	0.99	−32.81	0.24 ± 0.05	0.06 ± 0.02	0.38 ± 0.03	0.97	−31.30	0.27 ± 0.02
^177^Lu-DOTATATE	0.85	−34.25	0.03 ± 0.10	0.09 ± 0.07	0.16 ± 0.02	0.94	−43.20	0.06 ± 0.02

LQ = linear quadratic; AIC = Akaike information criterion; L = linear.

*R*^2^ and AIC are reported to evaluate best fit.

## DISCUSSION

Current clinical outcomes of PRRT underline the necessity for improved efficacy, which could be assisted by establishing dose–effect relationships through the integration of dosimetry and radiobiology (14). However, micrometastases, critical to disease eradication, pose significant challenges to dosimetry because of their undetectability. Furthermore, investigating PRRT-specific radiobiology is essential to revise EBRT-based dose–effect relationships (15). To this end, we characterized the biologic response of GOT1 and NCI-H69 cell lines to PRRT, exploring how physical heterogeneity and dose rate influence these responses compared with EBRT. To achieve this, dosimetry models were developed to reflect the exposure conditions of these cell lines during both viability (at day 7) and cell death (at multiple time points after PRRT), carefully assessing the impact of common simplifications in cellular dosimetry calculations. The natural tendency of these cell lines to form clusters enabled the creation of dosimetry models that could be clinically translated to micrometastases.

Although it has been shown that cellular morphology and proximity significantly affect the absorbed dose to the nucleus (15), this study introduces, to our knowledge, the first image-based dosimetry model capable of replicating realistic cellular placement in plated, cluster-forming cells without relying on time-consuming immunofluorescent staining or oversimplified symmetric assumptions typically adopted for β-emitting radionuclides (16,17). For suspension cells, for which precise placement is impossible to determine, we developed a method to account for cluster size variability by analyzing microscopy images at key time points, enabling more accurate average exposure calculations.

Our results indicate that in-suspension cellular models with similar experimental cell densities (∼200,000 cells/mL), leading to well-spaced cells and small clusters of 1–3 cells (GOT1, 0–4 h; NCI-H69, 0–24 h), can be realistically modeled by accounting for the contribution from the medium, the self-absorbed dose in a single cell, and the in-cluster cross-absorbed dose from 2–3 cells, without needing to account for subcellular radioactivity localization or specific cell placement within the cluster.

For fast-proliferating NCI-H69 cells, which remain in suspension after incubation, we modeled randomly growing configurations formed from closely seeded cells. Their larger size (7 vs. 5.5 µm in GOT1) and less efficient cluster packing caused cross-dose saturation to occur at smaller cluster sizes (20 cells) and at lower levels than in GOT1 (60 cells). Because of early cross-dose saturation and rapid activity excretion (effective half-life, 22.6 ± 0.15 h), most of the absorbed dose was delivered within 3–4 d, when clusters primarily formed through proliferation, making imaging of larger clusters at later stages less critical than modeling proliferation dynamics. The limited cross-absorbed dose and similar uptake values across activity concentrations resulted in minimal absorbed dose differences at day 7, with the medium slightly amplifying these at higher activities (e.g., 1 MBq/mL, 1.45 ± 0.19 to 1.78 ± 0.19 Gy).

For GOT1 cells, microscopic imaging in the plated condition revealed significant absorbed dose heterogeneity because of variable cluster sizes and cell placement, underscoring the limitations of traditional models with simplified geometries. Neglecting 2-dimensional cross-cluster contributions and cluster shape complexity underestimated the *S* value by up to 90% and failed to accurately capture heterogeneity, with initial dose rates varying up to 2.3 times within the same setup and absorbed doses ranging from 2.1 to 8.2 Gy over 7 d at 1 MBq/mL. Although receptor binding heterogeneity minimally affected the average absorbed dose, decreasing activity in the cluster center reduced the average dose but did not affect relative heterogeneity, which spanned a 4-fold range (Supplemental Fig. 11). These findings underscore that physical factors, such as irradiation geometry, are as important as biologic factors, such as receptor expression, in causing absorbed dose heterogeneity in vitro, especially in cell lines with relatively uniform receptor expression.

Although receptor saturation limited our ability to assess the absorbed dose response across the same range of high absorbed doses used for EBRT, the α-to-β ratios for NCI-H69 cells revealed distinct radiobiologic responses to EBRT and PRRT. For rapidly proliferating NCI-H69 cells, the difference in α-to-β ratios between EBRT (4 ± 1.57 Gy) and PRRT (0.33 ± 1.13 Gy) is quantified by the inverse of the Lea-Catcheside factor (1/G) of approximately 12, reflecting how the continuous low-dose delivery of PRRT alters the quadratic term’s contribution through a combination of sublethal damage repair, reoxygenation, redistribution, proliferation, and other dynamic processes that can enhance or diminish its effect (18). In addition, the maximum RBE for loss of viability, obtained by linearizing the dose response, was 0.43 ± 0.07 for PRRT compared with EBRT. This aligns with in vivo survival outcomes observed after PRRT exposure in our previous study (19) and prior findings reported for DLD-1 colorectal cancer cells treated with ^90^Y (maximum RBE for clonogenic survival, 0.4) (20).

For GOT1 cells, which represent the indolent behavior of NETs with a slow proliferation rate (doubling time, 18 d) (21), this study provides, to our knowledge, the first clinically relevant in vitro radiobiologic comparison between EBRT and PRRT. Both treatments followed linear dose–response models, indicating either a high α-to-β ratio or efficient sublethal damage repair, with RBE of 0.22 ± 0.02. This lower RBE is consistent with clinical observations that PRRT requires roughly 4 times higher absorbed doses than EBRT to achieve similar effects (22), likely due to the cells’ efficient repair mechanisms and slower proliferation. It is essential to establish RBE for relevant tumor cell lines such as GOT1, particularly given the potential local control benefits of adjuvant EBRT in patients with pancreatic NETs (22,23).

Assessment of both viability and cell death provides a more thorough evaluation of PRRT’s radiobiologic effect on cells. In NCI-H69 cells, EBRT induced significant cell death at a 2-Gy threshold, but PRRT, with RBE of 0.43, would require about 4.6 Gy to reach this effect. This threshold was not reached in our experiments, explaining the lack of significant instant cell death observed with PRRT. For GOT1 cells, at 7 d, approximately 29% of the total absorbed dose from ^177^Lu was still to be delivered (Supplemental Table 7), despite already reaching an efficacious dose rate of 9–15 mGy/h (Figs. 4A and 4B). Lower initial dose rates caused a more pronounced reduction in live cells, suggesting benefits of fractionated or hyperfractionated PRRT compared with single-dose regimens (24). Although this study provides insights, limitations remain in the radiobiologic modeling. The empiric models used do not explicitly account for low dose rate effects, including sublethal DNA damage dynamics, adaptive responses, bystander effects, or dose-dependent cell cycle redistribution changes (25). Moreover, the impact of prolonged low dose rate exposure on specific cell death pathways, such as apoptosis or senescence, requires further investigation. Additional experiments are needed to inform future mechanistic models.

## CONCLUSION

Accurate dosimetric assessment is essential for understanding PRRT’s biologic effects and evaluating its efficacy. Challenges in establishing clinical dose–response relationships arise from limited radiobiologic insights into PRRT’s prolonged and heterogeneous response, as well as significant uncertainties in dose calculations for smaller volumes. Our image-based multicellular dosimetry model addresses these challenges preclinically by replicating cross-irradiation in cluster-forming cells and incorporating critical parameters such as dose rate. This model enabled the establishment of dose–response relationships for NCI-H69 cells that align with in vivo behavior and provided clinically relevant RBE for GOT1 cells, highlighting the impact of dose rate levels. These findings support the potential translatability of this approach and underscore the importance of detailed dosimetric methods to advance PRRT’s clinical application.

## DISCLOSURE

This work was supported by an ERC grant (101042537 RADIOBIO). No other potential conflict of interest relevant to this article was reported.

## Notes

KEY POINTS **QUESTION:** How do radiobiologic parameters for PRRT compare with those for EBRT in clinically relevant neuroendocrine cancer cell models? **PERTINENT FINDINGS:** The 2-dimensional and 3-dimensional dosimetry models for cluster-forming cells, appropriate for micrometastases, indicate that PRRT requires 3–4 times the absorbed doses of EBRT to achieve similar effects. Significant differences in dose–response dynamics for cell death between PRRT and EBRT are evident and depend on cellular proliferation rates. **IMPLICATIONS FOR PATIENT CARE:** Our findings provide insights into dose rate thresholds for cell death in PRRT and can enable the combination of PRRT with EBRT, enhancing treatment outcomes.
